# Established and Emerging Asthma Biomarkers with a Focus on Biologic Trials: A Narrative Review

**DOI:** 10.3390/jpm15080370

**Published:** 2025-08-13

**Authors:** Philip F. Lavere, Kaitlin M. Phillips, Nicola A. Hanania, Muhammad Adrish

**Affiliations:** Section of Pulmonary, Critical Care, and Sleep Medicine, Baylor College of Medicine, Houston, TX 77030, USA; philip.lavere@bcm.edu (P.F.L.); kaitlin.phillips@bcm.edu (K.M.P.); hanania@bcm.edu (N.A.H.)

**Keywords:** biomarkers, asthma endotypes, asthma remission, IgE, FeNO, blood eosinophils, omalizumab, mepolizumab, benralizumab, dupilumab, tezepelumab

## Abstract

Chronic airway inflammation with variable airflow obstruction is clinical asthma, and it arises from distinct molecular and pathological mechanisms called endotypes. Biomarkers allow for precise endotype characterization and have been used in clinical trials to design, monitor, and evaluate outcomes for asthma biologic therapies. This review will highlight the central and evolving role of biomarkers for past, present, and future asthma, with a focus on regulatory-approved biologic therapies and emerging biomarkers. Established biomarkers, including serum immunoglobulin E (IgE), blood eosinophils, the fraction of exhaled nitric oxide (FeNO), and serum periostin, helped elucidate the complex pathophysiology of the eosinophilic type 2 (T2) asthma endotype. Emerging biomarkers, or older biomarkers with emerging utility, include sputum inflammatory cells (eosinophils, neutrophils, interleukins), thymus and activation-regulated chemokine (TARC), plasma eotaxin-3, eosinophil peroxidase (EPX), Clara/club cell secretory protein (CC16), and quantitative computerized tomography (QCT) imaging biomarkers (evaluating mucus plugging, air trapping, airway wall thickness, small airway remolding) and are increasingly used in clinical trials as secondary endpoints in evaluating efficacy, as well as in the clinical setting at specialized centers. The rapid advances in asthma research, due in part to biomarkers and biologic therapies, may soon standardize an end goal: symptom-free asthma remission without exacerbations.

## 1. Introduction

Biomarkers facilitate a personalized medicine approach and thus more specific and effective treatment plans. The U.S. FDA defines molecular biomarkers as characteristic indicators of biological or pathogenic processes, responsive to exposures or interventions (Table 1, [1]). Chronic airway inflammation with variable airflow obstruction represents the clinical asthma syndrome, which arises from distinct molecular mechanisms now defined by endotypes. With the help of biomarkers, numerous clinical trials have established targeted biologic therapies for the eosinophilic T2 (T2) asthma endotype. The impact of biomarkers has been substantial, with the 2020 European Respiratory Society/American Thoracic Society (ERS/ATS) guidelines for severe asthma providing three new biomarker-specific recommendations for clinical practice, compared to 2014, where only one biomarker recommendation was made for experienced centers [2,3]. The updated Global Initiative for Asthma (GINA) strategy highlights four biomarkers, particularly as they define the underlying endotype and help plan biologic therapy for severe asthma [4]. We believe expansive biomarker applications are on the horizon for asthma, and this narrative review will highlight the central and evolving role of biomarkers for past, present, and future asthma biologic therapy.

## 2. The Eosinophilic T2 Inflammatory Asthma Endotype

**Figure 1:**
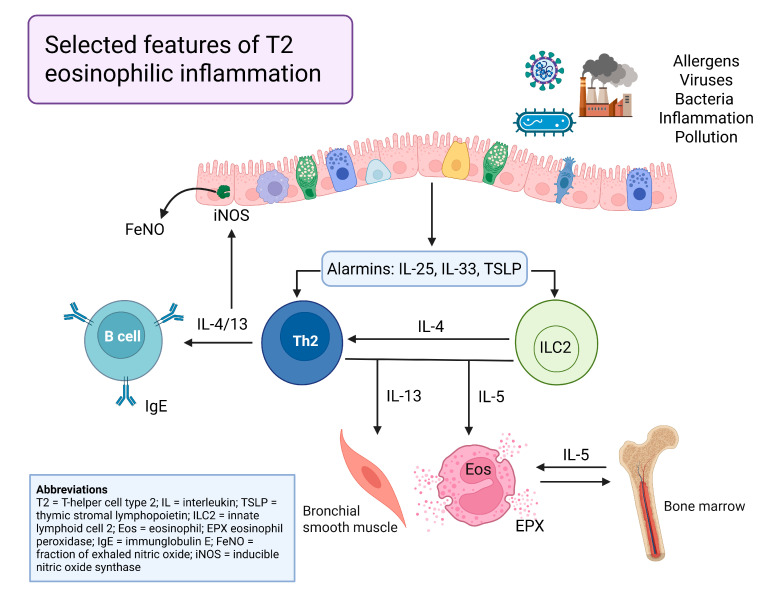
Key highlights of T2-mediated inflammation in eosinophilic asthma [5,6,7]. (created with biorender.com).

When the respiratory epithelium is exposed to stress, environmental pollutants, viruses, and bacteria, the epithelial cytokines (alarmins), including thymic stromal lymphopoietin (TSLP), interleukin 25 (IL-25), and interleukin 33 (IL-33), activate downstream cells, including the innate lymphoid cells (ILC2) [5,6,7]. (Figure 1) In turn, one function of ILC2s is augmenting the type-2 helper T-cell (TH2) cytokine inflammatory signature via interleukins 4, 5, and 13 (IL-4, IL-5, IL-13) [5,6,7]. Downstream effects include the following: (a) IL-4 signals TH2 cells to produce IL-5 and IL-13; (b) IL-4 encourages B-cell class switching and IgE production; (c) IL-5 stimulates bone marrow for eosinophil recruitment to the airway; (d) eosinophils make more IL-5 and IL-13, and many other cytokine and cellular recruitment pathways are activated [5,6,7].

## 3. Established Biomarkers Investigated in Biologic Clinical Trials

### 3.1. Serum Immunoglobulin E (IgE)

Before any asthma biologic trial, serum IgE was an established diagnostic biomarker for allergic asthma, and in children, it was a biomarker that was strongly associated with future development of asthma [8]. It was also well established that IgE-mediated immune pathways were central to asthma, which made serum IgE an intuitive choice to be the first biomarker featured in a biologic clinical trial for asthma (the anti-IgE therapy, omalizumab) [9]. Four large randomized controlled trials studied omalizumab vs. placebo in allergic asthma using serum IgE as part of their inclusion criteria, and all demonstrated significant reduction in asthma exacerbations, inhaled corticosteroid use, and improvement in symptom burden [10,11,12,13]. Two trials reported pharmacodynamic biomarker response to omalizumab with substantial reductions in serum free IgE by 89–99% [10,11]. However, the susceptibility and the diagnostic and pharmacodynamic response functions of serum free IgE did not translate into other biomarker domains (Table 2). Follow-up analysis of multiple trials largely found that baseline serum IgE levels do not predict clinical response to omalizumab, with perhaps one exception in the INNOVATE trial [14,15]. However, the EXTRA trial found that other type 2 inflammatory biomarkers were predictive of omalizumab’s ability to reduce asthma exacerbations (blood eosinophils, FeNO, serum periostin) [16].

Given the limitations of serum free IgE in predicting clinical response, other markers of T2 inflammation were favored in subsequent trial design, subgroup analysis, and secondary endpoints. Anti-IL5/IL5R trials rarely used IgE for their inclusion criteria or secondary outcomes [17,18,19,20,21]. Trials of the anti-IL4Rα biologic dupilumab featured the biomarkers of blood eosinophils and FeNO but also collected baseline serum IgE levels that ranged from 410 to 450 IU/mL [22,23,24]. Long-term dupilumab safety and efficacy extension studies noted sustained IgE reductions to 75 IU/mL, alongside improved lung function, better asthma control, and lower rates of exacerbation [25]. One small real-world cohort found patients with baseline IgE levels > 167 IU/mL had greater benefits with dupilumab [26]. Secondary endpoints of trials of the anti-TSLP Tezepelumab examined outcomes in T2-high vs. T2-low groups and found benefits across asthma endotypes defined in part by serum IgE levels > 100 IU/mL (T2 high) or < 100 IU/mL (T2 low) [27,28]. Taken together, these results make serum IgE a consistent and multifaceted asthma biomarker that is easily measured, cost-effective, and widely available.

### 3.2. Blood Eosinophils

Elevated blood eosinophils reflect T2 inflammation and are fundamental to asthma pathology. A large UK study found that for each 100 cell/µL increase in blood eosinophils, the relative risk of severe exacerbations rises steadily alongside decreasing odds of asthma control [29]. Indeed, another study found that the exacerbation incident rate ratio increased by 9% for each 100 cell/µL increase in blood eosinophils [30]. The French COBRA cohort over nine years found that increasing blood eosinophils over time was associated with exacerbations and diminished asthma control scores [31]. As a biomarker, it is cost-effective, widely available, and easily obtainable; it helps identify the eosinophilic asthma endotype, prognosticates exacerbations, and predicts clinical response to certain biologic therapies. There are, however, several limitations for this biomarker, as outlined in Table 3.

#### 3.2.1. Blood Eosinophils in Anti-IL5/IL5R Biologics (Mepolizumab, Benralizumab, Reslizumab)

The anti-IL5 mepolizumab initially had two negative randomized trials [32,33]. Subsequent trials focused on patients with severe asthma with elevated blood eosinophils ranging from ≥150–300 cells/µL and demonstrated a dramatic reduction in asthma exacerbations, improvement in lung function, and asthma-specific quality of life [17,18,34,35]. Open-label extension cohorts demonstrated sustained reductions in blood eosinophils, exacerbation rates, systemic corticosteroid requirements, and improvements in asthma symptom scores [36,37,38,39]. Nuanced blood eosinophil biomarker dynamics were observed in the mepolizumab extension cohorts. Furthermore, patients assigned to placebo in the MENSA and SIRIUS trials who were enrolled in the follow-up COSMOS study, in which they received mepolizumab, experienced a reduction in blood eosinophils from 280 to 60 cells/µL, alongside clinical improvement, for 52 weeks [37]. On trial completion, this cohort reported worsening asthma symptom scores within 12 weeks of discontinuing mepolizumab, with a concomitant rise in blood eosinophil counts to 160 cells/µL [37]. Over time, patients with severe asthma from MENSA and SIRIUS who did not enroll in COSMOS became a real-world mepolizumab interruption group, which was then studied in the COSMEX trial; these patients experienced worsening lung function, asthma symptoms, and an elevation in blood eosinophils (260 cells/µL), which all resolved when they were started back on mepolizumab [38]. The COMET trial assessed patients on mepolizumab for >3 years and randomized 1:1 to mepolizumab versus placebo. Notably, three years of mepolizumab treatment attained baseline blood eosinophils of 50 cells/µL, compared to 270 cells/µL for the placebo arm, who also suffered from faster asthma exacerbations and decreased asthma control [39].

Further research with the anti-IL5 biologics benralizumab and reslizumab followed a similar trial design, focusing on severe eosinophilic asthma defined by elevated blood eosinophil counts [19,20,21,40,41,42,43,44]. The cumulative biomarker data from these trials followed a similar theme: patients with moderate to severe eosinophilic asthma and elevated baseline blood eosinophils received anti-IL5 therapy; blood eosinophils quickly dropped, then exacerbation rates decreased, asthma control improved, total steroid dose decreased, and quality-of-life metrics improved. The benralizumab extension study, which included patients from SIRROCO and CALIMA, included roughly 1500 patients, where nearly 1000 patients with blood eosinophils > 300 cells/µL did not have an exacerbation across 2 years [45]. This remarkable observation was confirmed in a five-year extension study when exacerbations were eliminated in 60% of patients who had initial blood eosinophils > 300 cells/µL while on medium-to-high-dose inhaled corticosteroids, opening the possibility of blood eosinophils serving as a biomarker for “asthma clinical remission” on anti-IL-5 therapy [46]. Not surprisingly, real-world cohorts confirm elevated blood eosinophils >300 cells/µL are predictive of complete treatment response to anti-IL5/5R therapy [47].

Taken together, blood eosinophils constitute an excellent biomarker for anti-IL5 therapy fulfilling diagnostic endotyping, disease control monitoring, and pharmacodynamic response functions. The long-term extension trials suggest elevated blood eosinophils are prognostic of worsening asthma control and can clinically predict exacerbations.

#### 3.2.2. Blood Eosinophils in Anti-IL4Rα Biologic Trials (Dupilumab)

A phase 2 trial enrolled 100 patients with moderate-to-severe eosinophilic asthma (blood eosinophils > 300 cell/µL or sputum eosinophils > 3%) comparing dupilumab versus placebo for 12 weeks and found reduced exacerbations and improved secondary biomarker profiles (IgE, FeNO, TARC), with the notable finding that blood eosinophils sometimes increase on anti-IL4Rα therapy [22]. The follow-up phase 2 trial randomized patients with eosinophilic asthma by blood eosinophils greater or less than 300 cells/µL to dupilumab versus placebo and demonstrated reduced exacerbations and improved lung function regardless of initial blood eosinophil levels [48]. Larger phase 3 trials replicated these results over 52 weeks, again showing a transient rise in eosinophil levels on dupilumab, yet with significant reductions in exacerbations and improved asthma control [23,24]. Subgroup analysis revealed that patients with elevated baseline blood eosinophils enjoyed greater benefits with dupilumab [23,24]. The extension study found transient elevation in blood eosinophil level in prior placebo patients starting dupilumab that normalized at 48 weeks, with total reductions in blood eosinophils by 96 weeks [25]. 

Dupilumab has been linked with eosinophilia treatment emergent adverse events (TEAE) in clinical trials. Post hoc analysis of 11 large clinical trials involving dupilumab for asthma, atopic dermatitis, or chronic rhinosinusitis with nasal polyps found that in over 6000 patients on dupilumab, the incidence of eosinophilic TEAEs ranged from 0 to 13.6% [49]. Even so, clinical symptoms were rarely observed, and only a few patients exceeded blood eosinophils of >3000 cells/µL [49]. Eosinophilia on dupilumab is likely related to downstream cytokine effects from IL-4 and IL-13 blockade. Specifically, eosinophil migration into tissues is mediated by vascular cell adhesion molecule (VCAM) 1, and VCAM-1 is upregulated by IL-4 [50]. As blocking IL-4 and IL-13 does not influence eosinophil maturation and release from the bone marrow, dupilumab likely traps eosinophils in the blood compartment, which in some patients will manifest eosinophilia [49,50].

#### 3.2.3. Blood Eosinophils in Anti-TSLP Biologic Trials (Tezepelumab)

In a phase 2 trial of patients with uncontrolled moderate-to-severe asthma randomized to different doses of tezepelumab versus placebo, anti-TSLP therapy reduced asthma exacerbations regardless of high or low blood eosinophil subgroups [27]. In a global population with mixed asthma severity, tezepelumab reduced exacerbations and improved secondary endpoint inflammatory biomarkers across T2-high and T2-low groups, with a stronger effect size noted in T2 asthma endotypes [28]. Results from the DESTINATION extension study suggest higher baseline blood eosinophil counts were associated with greater likelihood of clinical asthma remission on tezepelumab [51]. The SOURCE and ARRIVAL trials are ongoing open-label extension studies designed to evaluate tezepelumab’s ability to reduce background inhaler and/or corticosteroid use, with secondary biomarker endpoints focused on blood eosinophils and FeNO [52,53].

### 3.3. Fraction of Exhaled Nitric Oxide (FeNO)

T2 inflammatory cytokines (mainly IL-13) stimulate inducible nitric oxide synthase in the respiratory epithelium to produce nitric oxide, which amplifies the inflammatory response [54]. Thus, fractional exhaled nitric oxide (FeNO) directly reflects type 2 airway inflammation. This biomarker has diagnostic, prognostic, predictive, and pharmacodynamic properties. It is a point-of-care test that is easily measured. However, there are several limitations for this biomarker measurement, as outlined in Table 3.

#### 3.3.1. FeNO in Anti-IgE and Anti-IL5/IL5R Biologic Therapies

There is varying evidence regarding baseline FeNO predicting clinical response to therapy with the anti IgE omalizumab. Specifically, patients from the EXTRA trial on omalizumab with high FeNO ≥ 19.5 ppb had a nearly three-fold reduction in asthma exacerbations compared to patients with low FeNO < 19 ppb (53% vs. 16%) [16]. FeNO was also suggested as a prognostic biomarker, as more patients in the FeNO-high placebo subgroup experienced asthma exacerbations compared to the FeNO-low placebo subgroup [16]. However, the 48-week, real-world, open-label prospective PROSPERO study found that over 600 patients responded well to omalizumab, with improvement in asthma control and exacerbation rates regardless of baseline FeNO levels [55].

Most anti-IL5/5R biologic trials did not systematically measure FeNO or target FeNO dynamics in their secondary outcomes; therefore, limited prospective data exist regarding FeNO’s biomarker performance within this biologic class. One exception is the DREAM study, which found comparable mepolizumab outcomes for FeNO ≥ 50 ppb versus < 50 ppb, suggesting FeNO is not predictive of mepolizumab clinical response [17]. A single-center retrospective analysis found no positive differences between baseline FeNO and anti-IL-5/5R monoclonal antibody (mAb) outcomes [56]. Another retrospective single-center cohort found no useful FeNO trends relating to clinical response on anti-IL-5/5R mAb therapy [57]. Current data therefore suggests FeNO is not a useful biomarker for anti-IL-5/5R mAb therapy, and it does not serve predictive and pharmacodynamic functions within this therapeutic class.

#### 3.3.2. FeNO in Anti-IL-13 mAb Therapy (Tralokinumab and Lebrikizumab)

Tralokinumab is a mAb against IL-13. In the phase 2 MESOS trial, tralokinumab reduced FeNO but did not alter airway eosinophilic inflammation as measured by blood and sputum eosinophils [58]. Indeed, two sequential large phase 3 clinical trials did not establish the utility of FeNO in anti-IL-13 biologic therapy; although high FeNO subgroups enjoyed significant reductions in annualized asthma exacerbations in STRATOS1, this finding was not replicated in STRATOS2 [59]. Lebrikizumab is an IgG4 mAb with high affinity against IL-13 but failed to show efficacy in one of two large phase 3 trials in adults with moderate to severe uncontrolled asthma [60]. However, post hoc pooled analysis of LAVOLTA I and II suggests that patients with a history of asthma exacerbations and FeNO > 50 ppb experienced almost 50% lower exacerbation rates on lebrikizumab than placebo [61]. FEV1 also significantly improved in the same subgroup of patients [61].

#### 3.3.3. FeNO in Anti-IL4Rα mAb Therapy

Post hoc analysis of the QUEST trial found the risk of asthma exacerbations could be stratified by FeNO; >50 ppb had 1.5 greater risk than in patients with <25 ppb and blood eosinophils < 150 cells/µL, and FeNO between 25 and 50 ppb had 1.3-fold greater risk than in patients < 25 ppb and blood eosinophils < 150 cells/µL. Composite biomarker data with FeNO ≥ 25 ppb and blood eosinophils ≥ 150 cells/µL was associated with a 3.6-fold greater exacerbation risk than in patients with FeNO < 25 ppb and blood eosinophils < 150 cells/µL. FeNO is a biomarker capable of prognosticating future asthma exacerbations in patients on anti-IL4Rα therapy [62]. The same stratified baseline FeNO ranges (<25, 25–50, >50 ppb) predicted clinical response to dupilumab with greater reductions in exacerbation risk (22%, 58%, and 69%, respectively) independent of blood eosinophil levels [63]. Conversely, additional post hoc analysis of QUEST found changes in FeNO were not associated with annual exacerbation rates, but FeNO reductions on dupilumab predicted improvements in lung function [64]. It is interesting to note that patients with baseline FeNO < 25 ppb did not benefit from dupilumab [23].

The phase 3 VENTURE trial confirmed the oral corticosteroid-sparing effects of dupilumab and again noted fewer exacerbations and improved lung function for the subgroup of patients with the highest baseline blood eosinophils and FeNO [24]. Suppressed FeNO < 25 ppb with improved airway volumes was the featured endpoint in the recent phase 4 VESTIGE trial, where dupilumab reduced FeNO versus placebo (57% vs. 11%), with improvements in airway volumes and mucus plugging [65].

#### 3.3.4. FeNO in Anti-TSLP mAb Therapy

A broader range of biomarkers were retrospectively studied in anti-TSLP clinical trials; T2 inflammatory markers, including FeNO, were correlated with downstream cytokines IL-5, IL-13, and periostin in the PATHWAY cohort [27,66]. Tezepelumab was more efficient in reducing downstream cytokines in patients with composite high levels of blood eosinophils and FeNO but, notably, it reduced exacerbations regardless of all measured baseline biomarkers. Tezepelumab’s broad efficacy across T2-high and -low endotypes was confirmed in the phase 3 NAVIGATOR trial, where 40% of participants had FENO values < 25ppb, and 58% had blood eosinophils < 300 cells/µL [28]. The 52-week NAVIGATOR extension study (DESTINATION) found higher baseline blood eosinophils and FeNO were associated with greater chances of clinical remission on tezepelumab, tapping both T2 biomarkers as potential predictors of anti-TSLP mAb therapy [51].

The post hoc biomarker analysis of NAVIGATOR and DESTINATION again noted that higher baseline blood eosinophils and FeNO were predictive of clinical remission on tezepelumab [67]. However, these biomarkers were reduced during the trial period even in patients who did not achieve clinical remission, highlighting their pharmacodynamic and monitoring functions but diminishing their capability as surrogate response and/or clinical predictive biomarkers for anti-TSLP treatment [67]. Although tezepelumab did not demonstrate an oral corticosteroid-sparing effect in the SOURCE trial, it again confirmed significant reductions in T2 biomarkers, including FeNO, alongside improved lung function and asthma control [52]. The phase 3 ARRIVAL trial is currently enrolling patients with severe uncontrolled asthma to evaluate tezepelumab’s ability to reduce maintenance therapy without loss of asthma control, defined by symptoms and biomarkers, and could provide additional data for FeNO as a biomarker in severe asthma [53].

#### 3.3.5. FeNO Applications from the Guidelines Across Levels of Asthma Severity

FeNO applications are varied across several society guidelines, highlighting its complexity as an asthma biomarker. Situated within its proper contextual interpretation, ATS and the United Kingdom National Institute for Health and Care Excellence (NICE) recommend FeNO for supporting a clinical diagnosis of asthma [68,69]. GINA notes FeNO applications throughout their recommendations, but most especially for endotyping severe asthma, assessing for residual airway inflammation in patients on high-dose inhaled or oral corticosteroids, monitoring inhaled corticosteroid adherence prior to biologics, tapering oral corticosteroids, and for initiating the biologics dupilumab and tezepelumab [4]. A small cross-sectional real-life study found FeNO’s correlation with asthma exacerbations at 72% sensitivity and 79% specificity [70]. As a prognostic biomarker in severe asthma, elevated FeNO predicts worsening asthma control, lung function decline, and increased risk of exacerbations. The relative ease and point-of-care nature of FeNO testing, alongside its affordability, makes it an excellent biomarker for eosinophilic asthma in clinical practice today.

### 3.4. Serum Periostin

Serum periostin is generated by airway epithelial cells in response to IL-13 and is a marker of T2-related airway inflammation in patients with asthma [71,72]. Patients with high baseline periostin demonstrated a greater improvement in lung function on anti–IL-13 therapy compared with those with low periostin levels [73]. Further, two phase 2b trials of lebrikizumab versus placebo in moderate to severe asthma found periostin-high patients (≥50 ng/mL) experienced a 60% reduction in asthma exacerbations and a 9% forced expiratory volume in one second (FEV1) improvement compared to periostin-low patients (<50 ng/mL) [74]. However, as in the case of elevated FeNO, the LAVOLTA I and II trials did not demonstrate that periostin-high patients clinically benefit from lebrikizumab, confirming it does not currently function as a clinical response biomarker for anti-IL-13 therapy [60].

The 52-week ARIETTA prospective study enrolled over 460 patients to examine clinical predictive and prognostic T2 biomarkers for severe asthma. The primary endpoint was the asthma exacerbation rate in periostin-high (≥50 ng/mL at baseline) versus periostin-low (<50 ng/mL) groups, which was not significant. Periostin was not found to be a prognostic biomarker for severe asthma exacerbations. Like several other trials, composite elevations in blood eosinophils ≥ 300 cells/μL and FeNO ≥ 25 ppb were associated with higher exacerbation rates [75]. It should be noted that systemic steroids, co-morbid allergies, and dietary patterns can alter blood eosinophils and/or FeNO values, so these confounders should be kept in mind when interpreting their use as biomarkers.

## 4. Emerging Biomarkers Across Asthma Endotypes

### 4.1. Sputum Markers

#### 4.1.1. Airway Eosinophils/Granule Contents

Sputum biomarkers may further characterize asthma into T2-high and T2-low endotypes. Sputum eosinophilia (≥3% of total sputum cells) is a well-described marker of T2 airway inflammation and is a clinical predictive biomarker for asthma biologic therapies [29]. Patients with elevated sputum eosinophils respond markedly well to mepolizumab and benralizumab, with decreased frequency of exacerbations and improved daily asthma control and lung function [76]. Mepolizumab notably decreases sputum eosinophil levels from baseline, and patients receiving mepolizumab in an acute exacerbation exhibit lower sputum eosinophilia with more mild exacerbation features compared to placebo patients during exacerbation [17,77]. Measuring sputum eosinophils may identify patients with severe asthma dependent on oral corticosteroids who could achieve steroid-free control on mepolizumab [78]. Unlike anti-IL-5/IL-5R biologics, omalizumab has not been associated with a reduction in sputum eosinophils [79]. However, the CASCADE trial demonstrated that tezelepumab reduces airway eosinophil count in bronchoscopic biopsy samples, irrespective of blood eosinophil count [80]. Unfortunately, the use of sputum eosinophils in clinical practice is limited by institutional availability of the test and the invasive nature of the sputum induction process required to collect the sample. As such, most of its utility is currently in the research setting.

#### 4.1.2. Sputum Neutrophils

Assessing sputum neutrophils has potential to further identify and classify T2-low asthma. T2-low asthma currently has three main groups: neutrophilic asthma (sputum neutrophilia), mixed granulocytic asthma (both sputum neutrophilia and sputum eosinophilia), and paucigranulocytic asthma (neither sputum neutrophilia nor sputum eosinophilia present) [81,82]. Patients with neutrophilic and mixed granulocytic asthma have more features of severe asthma, including reduced lung function, poorer response to bronchodilators, and more complex medication regimens when compared to the other categories [82,83]. Neutrophilic asthma is more associated with older age, heavier smoking history, and increased rates of asthma–COPD (chronic obstructive pulmonary disease) overlap when compared to the other endotypes [84]. Some studies have linked increased sputum neutrophilia to increased inhaled corticosteroid use, a relationship theorized to be a result of steroid-induced inhibition of neutrophil apoptosis [85].

The C-X-C motif chemokine receptor (CXCR)2 (an IL-8 receptor) has been identified as a potential therapeutic target for T2-low asthma. Two CXCR2 inhibitors, AZD6059 and SCH 527123, have demonstrated a reduction in sputum neutrophils in clinical trials but have failed to demonstrate clinical benefit [86,87]. In a small cohort of 50 patients with asthma on ICS, the presence of sputum neutrophilic inflammation was associated with diffusion-dependent peripheral airways, whereas sputum eosinophilic inflammation was associated with convection-dependent airways, indicating that sputum inflammatory cell type may involve different airway regions [88]. Thus, sputum neutrophil count has some prognostic and predictive biomarker functionality, but there are no regulatory-approved anti-neutrophil biologic therapies currently, and progress is slow for T2-low asthma.

#### 4.1.3. Sputum Interleukins (4, 5, 8, 13, 33)

Sputum cytokines are a promising category of biomarkers in asthma management. A small cohort of patients treated with benralizumab demonstrated that patients with elevated sputum interleukin-5 were more likely to achieve better asthma control and lung function [89]. In the same cohort, patients with elevated sputum interleukins-6 and -8 (IL-6 and IL-8) were less likely to see this benefit [89]. Elevated sputum IL-6 levels are correlated with exacerbations of mixed eosinophilic and neutrophilic asthma and are associated with worse lung function [90]. A study assessing the role of the tumor necrosis factor-alpha (TNF-alpha) antagonist etanercept in refractory severe asthma monitored sputum IL-8 levels during treatment. While the treatment arm showed improvement in airflow obstruction and modest response in asthma quality-of-life scores, sputum IL-8 levels did not change and are thus unlikely to be relevant as a monitoring biomarker for these therapies [91].

Sputum IL-13 may be a helpful biomarker in assessing asthma control, as higher sputum IL-13 levels have been linked to poor asthma control. In certain patients with severe asthma, IL-13 levels in sputum and bronchial biopsy samples stay elevated despite treatment with both inhaled and systemic corticosteroids [92]. Lower sputum IL-13 levels have demonstrated better diagnostic accuracy than both sputum eosinophils and FeNO in identifying well-controlled asthma, suggesting a role as a monitoring biomarker in the assessment of asthma control [93]. Higher levels of sputum IL-13 also predict greater response to omalizumab [94]. High levels of sputum IL-33 are linked to mixed granulocytic asthma [95]. Patients with asthma who had higher capsaicin cough sensitivity, as well as those with functional dyspepsia, showed significantly higher sputum IL-33 levels compared to those without these features [96].

### 4.2. Novel Biomarkers

#### 4.2.1. Thymus and Activation-Regulated Chemokine (TARC)

Thymus and activation-regulated chemokine (TARC) is a promising biomarker for patients with severe asthma requiring biologic therapy [97]. TARC, also known as CCL17 (C-C chemokine ligand 17), is a chemokine that attracts T2 helper cells into tissues [98]. TARC levels are increased in bronchioalveolar lavage fluid after an endobronchial allergen challenge in allergic asthma patients [99]. Dupilumab binds IL-4Rα, which blocks IL-4 and IL-13 cytokines and decreases TARC levels and subsequent eosinophil tissue recruitment [97]. A phase 2 trial of dupilumab versus placebo in patients with uncontrolled moderate-to-severe asthma found significant reductions in TARC (roughly 30% average baseline around 500 pg/mL, decreased by 200 pg/mL) in secondary biomarker endpoints [22]. The phase 3 QUEST trial reported a roughly 23% decrease in TARC levels for patients with uncontrolled moderate-to-severe asthma on dupilumab [23]. A small biomarker study found TARC was decreased with corticosteroids after mepolizumab treatment [100].

In other T2-mediated inflammatory diseases like atopic dermatitis, dupilumab quickly reduced serum TARC levels, which was correlated with clinical improvement in disease features [101]. A more recent meta-analysis of dupilumab has been shown to reduce serum levels across multiple atopic/allergic disease states, including asthma [102]. In the PATHWAY trial, tezepelumab reduced TARC levels, but clinical response to tezepelumab was not necessarily associated with changes in serum TARC [27]. Anti-IL13 mAbs (tralokinumab and lebrikizumab) reduce TARC levels, but TARC data was not reported in the LAVOLTA I, LAVOLTA II, STRATOS1, or STRATOS2 trials, so there is minimal clinical data regarding anti-IL13 therapy and TARC biomarker utility for asthma [59,60,103]. It is clear that TARC has pharmacodynamic response functions for dupilumab that warrant further study, but currently there are no additional biomarker functions ready for clinical practice.

#### 4.2.2. Plasma Eotaxin-3

Increased IL-13 release induces an upregulated expression of plasma eotaxin-3, also known as C-C Motif Chemokine Ligand 26 (CCL26). Increased eotaxin-3 is another important chemokine in type 2 inflammation; levels in sputum have been associated with severe asthma and are associated with elevated sputum eosinophils [104]. In the same phase 2 trial where dupilumab reduced TARC, eotaxin-3 levels were significantly reduced on dupilumab compared to placebo [22]. This was replicated in the QUEST trial, where dupilumab rapidly suppressed eotaxin-3 by week 12, with over 30% reduction in eotaxin-3 levels by week 52 compared to placebo [23]. The MAPLE biomarker study found suppressed eotaxin-3 levels when patients took corticosteroids after mepolizumab treatment [100]. Overall, although included in some secondary endpoints in trials, eotaxin-3 seems to be a biomarker still in the research stage at this time.

#### 4.2.3. Eosinophil Peroxidase (EPX)

Activated eosinophils release eosinophil peroxidase as part of their inflammatory response. Serum EPX is linked to systemic eosinophilic inflammation, and sputum EPX proved to be an even more sensitive biomarker of eosinophilic airways than sputum eosinophils [105,106]. Specifically, a 3-year study following serum and sputum EPX levels in asthma patients found persistently high EPX levels associated with worse airflow obstruction and exacerbations [106]. This finding was notable because 27% of patients with blood eosinophils < 150 cells/µL had high sputum EPX levels, thereby identifying an additional subset of patients with T2-high inflammation likely to benefit from asthma biologic therapy that are not captured by traditional blood eosinophilia [106,107]. Further EPX biomarker dynamics were analyzed for a subset of almost 60 patients who initiated mepolizumab therapy during the observation period; nearly all (96%) had normalization of serum EPX levels, whereas less than half (49%) had normalization of sputum EPX levels [106].

Discordant EPX and blood eosinophil biomarkers have been observed in previous studies. A small cohort of patients with mild allergic asthma underwent allergen challenge and despite mepolizumab administration causing reduced numbers of airway eosinophils, subjects showed elevated levels of EPX bronchial tissue staining which indicates ongoing eosinophil degranulation activity [108]. This observation may explain why some patients on mepolizumab still experience asthma exacerbations [108]. Such persistent elevations of airway EPX may represent a particularly difficult-to-control severe asthma endotype, which seems to have autoantibodies against sputum EPX [109]. Another small study comparing fixed-dose oral mepolizumab with weight-based intravenous reslizumab showed greater improvements in airway eosinophilia and asthma control, with the notable observation that four of six mepolizumab non-responders had anti-EPX autoantibodies [110]. Although not used as widespread as other T2 high biomarkers, sputum EPX and anti-EPX autoantibodies may prove useful for severe asthma endotyping and biologic therapy in the future.

#### 4.2.4. Clara/Club Cell Secretory Protein (CC16)

The CC16 protein is expressed in non-ciliated bronchial epithelial cells and is a biomarker for epithelial integrity or injury in a broad range of airway diseases [111,112]. Low CC16 levels in bronchioalveolar lavage (BAL) is associated with developing asthma [113]. A small cohort of patients with asthma were given an allergen challenge and showed increased levels of CC16 in plasma within an hour [114]. When asthmatic mice were exposed to particulate matter 2.5, the administration of CC16 blunted sinorespiratory symptoms, airway reactivity, and numerous T2 cytokines, including IL-5, IL-6, and IL-13 [115]. In a multicenter study, the expression of CC16 mRNA in bronchial epithelial cells was analyzed in association with clinical asthma severity. Reduced CC16 expression was associated with increased T2 biomarkers, worsening lung function, and increased risk of asthma exacerbations [116].

A large case–control study examined 1200 patients with various asthma types compared to 2000 healthy controls to determine associations in CC16 genetic polymorphisms with adult asthma control—the authors identified one specific single nucleotide polymorphism of CC16 associated with uncontrolled asthma [117]. Indeed, another combined cohort analysis of over 4000 patients across childhood into adulthood found low CC16 levels were associated with asthma from childhood into adulthood [118]. A Japanese cohort of 120 patients with severe asthma showed serum CC16 was inversely correlated with sputum eosinophils and blood periostin levels, but that CC16 did not predict asthma exacerbations [119].

### 4.3. Imaging Biomarkers

#### 4.3.1. Mucus Plugging

Mucus plugs are more commonly observed on computed tomography (CT) in severe eosinophilic asthma and are linked to airflow obstruction [120]. Quantifying mucous plugs on CT may provide prognostic clinical utility, as a high mucous plug score (mucous plugs visible in >4 segments) is highly associated with moderate to severe pre-bronchodilator airflow obstruction by FEV1 [121]. Progression of CT evidence of airway mucous plugging is associated with worse asthma symptom control, increased exacerbation rates, advancing decline in lung function, and worsening airflow obstruction over time [122]. Some data suggests a relationship between mucous plugging and T2-high asthma, implying its role as an important imaging biomarker in phenotyping asthma. Mucous plugging is highly associated with sputum eosinophilia, with one study demonstrating that CT evidence of mucous plugging was present in 100% of asthma patients with sputum eosinophilia and 36% of those without sputum eosinophilia [121,123]. This same study associated CT evidence of mucous plugging with elevated FeNO [123]. The biologic therapies benralizumab, dupilumab, and tezepelumab reduce mucous plugging on imaging, which may be linked to their therapeutic efficacy [65,124,125]. Monitoring of mucous plugging on CT may be a promising monitoring biomarker of therapeutic response to biologic therapies, as improvement in mucous plugging on CT is associated with improved FEV1 and asthma symptom scores.

#### 4.3.2. Air Trapping

Air trapping on pulmonary function tests (increased residual volume, increased residual volume/total lung capacity) can occur in asthma, and these spirometry indices have CT correlates that are increasingly relevant. Increased air trapping segment scores have higher rates of severe asthma, increased blood and sputum eosinophilia, and mucus plugging in the airways [126,127]. Air trapping on CT is associated with postbronchodilator FEV1 < 80% predicted and is one of the best imaging predictors of lung function in asthma [128]. Air trapping on CT is associated with prior asthma-related hospitalizations, including admission to the intensive care unit and the need for mechanical ventilation [129].

#### 4.3.3. Airway Wall Thickness

Airway wall thickness and total airway wall area are correlated with severe asthma by increased bronchodilator use, oral corticosteroid requirements, and worse airflow obstruction by FEV1 [130]. Average airway wall area is increased in both asthma and COPD when compared to control subjects, while the ratio of wall lumen area to body surface area is significantly lower in severe asthma patients [127]. While increased wall thickness is seen in severe asthma, there is no significant difference in airway wall thickness between mild–moderate asthma and control subjects [130]. In asthma patients with severe airflow limitation, the proximal airway lumen may increase, theoretically a compensatory response to advancing small airway disease [127]. There may be a relationship between imaging evidence of increased airway wall thickness and T2-high asthma, as it is associated with elevated blood eosinophilia [127,131]. Some data suggests that benralizumab treatment is associated with reduced wall thickness as well [132].

#### 4.3.4. Small Airway Remodeling

Imaging evidence of small airway remodeling is evolving as a prognostic imaging biomarker in asthma. Small airway remodeling is often seen in patients with severe asthma with tobacco smoking history. Patients with evidence of small airway remodeling on CT have been associated with severely reduced lung function with fixed airflow obstruction. These patients demonstrate higher utilization of controller therapies and corticosteroids [131]. Reduced total small airway count (≥10 missing sub-subsegmental airways) due to worsening small airway wall thickness and luminal narrowing is associated with worse asthma control and weakly correlated with reduction in FEV1 [133]. Airway smooth muscle cells contribute to airway remodeling with irreversible airflow obstruction and can demonstrate increased smooth muscle layer thickness on endobronchial biopsy. Specifically, the MELISCO study found that patients with severe eosinophilic asthma and a post-bronchodilator forced expiratory volume in one second to forced vital capacity (FEV1/FVC) ratio of <70% benefitted from 12 months of mepolizumab treatment, with improvements in pathologic airway smooth muscle indices [134].

#### 4.3.5. Hyperpolarized Gas Magnetic Resonance Imaging (MRI)

Hyperpolarized gas (Helium-3, xenon-129) is non-radioactive and can be inhaled for airspace volumetric mapping. Regional differences in ventilation and airflow obstruction are measured on MRI with high precision and can be used to better understand asthma at the individual level [135]. A small study of 27 patients found regional ventilation defects in patients with uncontrolled eosinophilic airway inflammation, suggesting heterogenous ventilatory defects are present in some patients with asthma [136]. Another small cohort of patients with severe steroid-dependent uncontrolled asthma and evidence of ongoing sputum eosinophilic inflammation were studied with hyperpolarized gas MRI before and after starting biologic therapy or increasing steroid dose. Significant improvements in regional ventilation defects measured by MRI were observed after escalation of therapy [137]. Additional larger studies are required, but hyperpolarized MRI offers a precise and highly individualized radiographic biomarker for severe asthma management.

## 5. Clinical Applications

### 5.1. Biomarkers in Society Guidelines

The 2014 ERS/ATS guidelines for severe asthma provided one conditional recommendation with very low evidence for biomarkers in asthma diagnosis: sputum eosinophils alongside the clinical impression can be used in experienced centers [2]. Given the available evidence at that time, ERS/ATS recommended against measuring FeNO to guide therapy, based on cost and unclear monitoring benefits [2]. At the same time, GINA did not comment on monitoring biomarkers.

Key changes to the 2020 ERS/ATS severe asthma guidelines highlight significant progress of biomarkers in asthma care. In 2025, ERS/ATS recommends that in adults with severe asthma and a history of exacerbation, measurements of blood eosinophils ≥ 150 cells/uL can be used to initiate anti-IL5 biologic therapy [3]. In 2019, GINA noted the utility of blood eosinophils ≥ 300 cells/µL for starting anti-IL5/5R therapy in patients with uncontrolled asthma on steps 4 or 5 [138]. Between 2014 and 2020, the ERS/ATS severe asthma guidelines newly recommended that the T2 biomarkers of blood eosinophils ≥ 260 cells/µL and FeNO ≥ 19.5 ppb can be used to identify adults with severe allergic asthma likely to benefit from anti-IgE therapy (alongside total IgE level) [3]. Since 2019, the GINA guidelines have noted that blood eosinophils and FeNO biomarkers may predict a favorable response to biologic therapy. Both updated ERS/ATS and GINA now recommend the anti-IL4Rα therapy dupilumab for adults with severe eosinophilic asthma dependent on oral corticosteroids [3,4]

### 5.2. Composite Biomarkers

A pooled analysis of seven biologic clinical trials involving over 5700 patients with severe uncontrolled asthma showed the composite biomarker of blood eosinophils ≥ 300 cells/µL and FeNO ≥ 50 ppb yielded severe exacerbation rates threefold higher than patients with blood eosinophils < 150 cells/uL and FeNO < 25 ppb. Of note in this study, baseline IgE did not prognosticate asthma exacerbation frequency [139]. The recent ORACLE2 meta-analysis obtained patient-level data from over 6000 people enrolled in asthma clinical trials and assessed T2 biomarkers for prognostic value in predicting asthma exacerbations. Elevated blood eosinophils and FeNO were synergistically predictive of increased exacerbation risk, confirming their composite biomarker utility in risk-stratifying asthma patients and future exacerbations [140].

The broad clinical utility of T2 endotyping exacerbations in obstructive lung disease was demonstrated in the phase 2 ABRA study. In almost 160 patients with physician-diagnosed asthma or chronic obstructive pulmonary disease, a history of exacerbation in the last 12 months, and a documented blood eosinophil level of >250 cells/µL, a single dose of benralizumab with or without prednisolone was compared to prednisolone only for “eosinophilic exacerbations.” Primary outcomes included treatment failure at 90 days and visual analog scale symptoms at 28 days. Pooled benralizumab groups showed significant reductions in treatment failure (45% vs. 74%) and improvements in symptom scores, highlighting the value of biomarkers defining T2 status with specific anti-T2 treatment implications [141].

Another notable biomarker study compared histopathologic scores from endobronchial biopsy versus a composite of T2 inflammatory markers (blood eosinophils and FeNO) to evaluate which system predicted response to biologic therapy. Response was defined by a composite measure of exacerbation rates, oral steroid use, asthma control, and FEV1. In over 60 patients with severe uncontrolled asthma who received anti-IL5/5R therapy, the baseline histopathologic score was a better predictor of response than T2 markers, indicating the potential benefits of tissue sampling to help guide therapy for patients with severe uncontrolled asthma [142].

Most often a single measurement of elevated blood eosinophils was used to help characterize asthma patients with T2 inflammation in biologic clinical trials; however, an individual’s blood eosinophils fluctuate across time and in different settings. The placebo arm from the clinical trials offers a unique opportunity to study eosinophil dynamics within patients across time in a controlled setting. Post hoc pooled analysis of two phase 3 reslizumab studies examined over 470 patients and found substantial variability in their blood eosinophil measurements [143]. Blood eosinophil variability was also demonstrated in over 2500 patients from placebo arms in four lebrikizumab trials [144]. Another retrospective analysis examined blood eosinophils, FeNO, and serum total IgE in over 240 patients and noted a wide variation in T2 inflammatory markers across time [145]. The use of composite biomarkers is useful to offset variable measurements of biomarkers across time.

## 6. Future Directions and New Research

### Biomarkers for Current Biologic Development

Like the six FDA-approved biologic therapies for asthma, emerging biologic therapies also rely on biomarkers for trial design, primary and secondary endpoints, proof of mechanism, and more. Biologics with ultra-long action, targeting additional cytokines, binding multiple antigens, or inhaled routes of delivery are all in early clinical trials [146]. Rademikibart is an anti-IL4Rα antagonist with a higher affinity than dupilumab that significantly improved FEV1 compared to placebo in a phase 2 trial with over 300 patients [147]. Like the QUEST trial, greatest effect size occurred in the subgroup of patients with the highest baseline blood eosinophils, with no significant effects for blood eosinophils < 150 cells/µL and FeNO < 25 ppb [147]. Depemokimab is an ultra-long-acting anti-IL-5 mAb given twice yearly that reduces asthma exacerbations for patients with severe eosinophilic asthma in the SWIFT1 and SWIFT2 phase 3 trials. FeNO was not reported, but there was a sustained reduction in blood eosinophils < 200 cells/µL across 52 weeks, and depemokimab was effective across all strata of serum IgE values [148]. Lunsekimig is a bispecific antibody “chain” constructed against TSLP and IL-13 that reduced FeNO, blood eosinophils, IL-5, eotaxin, IgE, and TARC after a single dose in a phase 1 trial [149].

Itepekimab is an anti-IL-33 biologic studied in combination with dupilumab in a phase 2 trial over 12 weeks for asthma control. All patients had severe asthma with baseline blood eosinophils > 300 cells/µL, baseline FeNO ranging from 24 to 33 ppb, and total IgE ranging from 320 to 680 IU/mL. Itepekimab reduced T2 asthma biomarkers more than placebo, but less than when combined with dupilumab. Yet it was observed that antagonizing IL-33 did not lower blood eosinophils as much as the established anti-IL5 mAb therapies, and that the IL-13 product eotaxin-3 was not suppressed as much as it was with dupilumab, leading the authors to speculate that anti-IL-33 therapy incompletely inhibits T2 inflammation, while other alarmins like TSLP are still pathologically active [150].

Tozorakimab is another biologic that blocks IL-33 and that was shown to improve lung function and reduce blood eosinophils and FeNO in patients with moderate-to-severe asthma in a 16-week phase 2 trial. Notably, almost 180 of the 235 patients (76%) had baseline blood eosinophils < 300 cells/µL, opening possible benefits for patients with a T2-low asthma endotype [151].

Astegolimab inhibits the IL-33 receptor (ST-2) and was studied in a phase 2 trial involving adults with uncontrolled severe asthma with the primary endpoint of annualized asthma exacerbation rates at 1 year. Patients on the highest dose of astegolimab met the significance level of reduced annual exacerbation rates compared to placebo. Subgroup analysis produced interesting results: eosinophil-low (<300 cells/µL) patients benefitted from reduced exacerbations compared to placebo, but this was not observed in the eosinophil-high group (≥300 cells/µL). Secondary biomarkers were assessed and although blood eosinophils were quickly and consistently suppressed, FeNO levels did not change compared to placebo [152]. Like tezepelumab, other biologic therapies targeting the alarmin pathway via anti-IL-33 activity show promise for patients with T2-low asthma, but more research in phase 3 trials is needed before regulatory approval.

## 7. Conclusions

Biomarkers have an emerging featured role in severe asthma care. On the horizon, biomarkers will continue to offer a more precision guide to biologic therapy in severe asthma. A recent editorial discussed the future of asthma and the possibility of using biologic therapy in clinically mild to moderate asthma, but with biomarker evidence of uncontrolled T2 inflammation, to prevent the development of severe asthma [153]. Such an approach would situate biomarkers in the center of all asthma therapy, delineating patients who are controlled, uncontrolled, and those achieving clinical remission. Further research into this domain is needed to advance our approach to asthma and improve patients’ outcomes.

## Figures and Tables

**Table 1:** FDA biomarker classifications and examples of applications in asthma.

Biomarker Type	Description	Example for Asthma
Susceptibility	Increased or decreased risk	Who develops asthma?
Diagnostic	Confirms disease	Classifying endotypes
Monitoring	Assesses disease status	Symptom control, airway remolding
Prognostic	Likelihood of clinical event or disease progression	Exacerbations, morbidity, mortality
Predictive	Favorable or unfavorable response to treatment	Symptom control, lung function
Response—pharmacodynamic	Biological response after therapy or exposure	IgE, FeNO, blood or sputum eosinophils
Response—surrogate endpoint	Predicts clinical benefit or harm	Exacerbations, airway remodeling
Safety	Detects or predicts exposure effects, toxicity, or need for treatment	Infections, morbidity, immunological alterations

Definitions: IgE = immunoglobulin E, FeNO = fraction of exhaled nitric oxide. Susceptibility is an increased or decreased risk of developing asthma; Diagnostic detects or confirms asthma endotypes; Monitoring assesses asthma status and response to treatment or exacerbating factors; Prognostic indicates increased or decreased likelihood of future clinical asthma events or progression; Predictive identifies favorable asthma endotypes for different treatments, including improved survival, or unfavorable asthma endotypes for certain adverse events, including mortality; Response biomarkers have two distinctions: (1) Pharmacodynamic follows biological response, either harmful or beneficial, in an asthma patient on different therapies or with different exposures, or (2) Surrogate endpoint biomarkers can predict clinical benefit or harm in clinical trials; Safety detects or predicts adverse drug or exposure effects for asthma patients, including toxicity, or necessity of treatment.

**Table 2:** Key biomarker applications for FDA-approved biologics.

Biological Agent	FeNO		Blood Eosinophils		Serum IgE	
	Response Predictor	Response Measure	Response Predictor	Response Measure	Response Predictor	Response Measure
Anti-IgE (Omalizumab)	+	↓	+	↓	No	No
Anti-IL5 (Mepolizumab, Benralizumab, Reslizumab)	No	No	+ + +	↓↓	No	No
Anti-IL4Rα (Dupilumab)	+ + +	↓↓↓	+ +	↑	No	↓↓
Anti-TSLP (Tezepelumab)	+ + +	↓↓↓	+ +	↓↓	No	↓↓

Definitions/Key: + through +++ minimal to maximal prediction of response; ↓ through ↓↓↓ minimal to maximal decrease; ↑ through ↑ ↑ ↑ minimal to maximal increase; IgE = Immunoglobulin E, FeNO = fraction of exhaled nitric oxide, IL = interleukin, TSLP = thymic stromal lymphopoietin.

**Table 3:** Strengths, limitations, and applications of selected asthma biomarkers.

Biomarker	Strengths	Limitations	Application
Serum IgE	- Minimally invasive - Easy to measure - Cost effective - Pharmacodynamic with biologics targeting IL-4	- Does not predict response to anti-IgE or other biologics - No prognostic characteristics	- May identify allergic phenotype - Associated with asthma severity and airway remodeling
Blood eosinophils	- Minimally invasive - Easy to measure - Cost effective - Widely measured - Correlates with sputum eosinophils - Predictive and prognostic properties - Pharmacodynamic with steroid therapy and biologics targeting IL-5	- Varying cutoffs to determine predictive characteristics - Multiple confounders (day-to-day variability, smoking, steroid therapy, parasitic infections, allergic diseases, eosinophilic conditions other than asthma)	- >150 cells/µL suggests T2 inflammation - >300 cells/µL is common threshold for biologic activity - Predicts exacerbations, poor asthma control, lung function decline, response to steroids and biologics
FeNO	- Minimally invasive - Easy to measure - Point of care	- Multiple confounders (smoking, allergen exposure, diet, corticosteroid use) - Cost and reimbursement issues - Day to day variability - Requires special equipment - Different cutoffs to determine high based on use and dose of ICS (naive > 50 ppb, medium dose ICS ≥ 25 ppb, high dose ICS ≥ 20 ppb)	- Identifies T2 airway inflammation - Predicts exacerbations, airway hyperreactivity and lung function decline - Predicts response to corticosteroids and biologics targeting IL-13
Serum periostin	- Marker of IL-13 activity and T2 airway inflammation	- Not currently used in the clinic setting - Can be elevated in growing children - Day to day variability	- Predicts a greater airway obstruction, exacerbation, and decline in lung function - Predicts response in biologics targeting IL-13
Sputum eosinophils	- Non-invasive - Reflects the lower airways	- Difficult to collect - Not all patients can provide samples - Complex processing - Requires specialized training, equipment and laboratory	- Defines T2 inflammatory phenotypes - Predicts exacerbation, poor asthma control, lung function decline, and response to steroids and biologics

Definitions: IgE = Immunoglobulin E, IL = interleukin, FeNO = fraction of exhaled nitric oxide, ICS inhaled corticosteroid, ppb = parts per billion.

## Data Availability

No new data were created or analyzed in this study. Data sharing is not applicable to this article.
